# Healthcare Costs Associated with Complications in Patients with Type 2 Diabetes among 1.85 Million Adults in Beijing, China

**DOI:** 10.3390/ijerph18073693

**Published:** 2021-04-01

**Authors:** Jun-Hui Wu, Yao Wu, Zi-Jing Wang, Yi-Qun Wu, Tao Wu, Meng-Ying Wang, Xiao-Wen Wang, Si-Yue Wang, Jia-Ting Wang, Huan Yu, Yong-Hua Hu

**Affiliations:** 1 Department of Epidemiology and Biostatistics, School of Public Health, Peking University, No. 38 Xueyuan Road, Beijing 100191, China; junhui@pku.edu.cn (J.-H.W.); yaowu@pku.edu.cn (Y.W.); wangzijing14@pku.edu.cn (Z.-J.W.); qywu118@163.com (Y.-Q.W.); twu@bjmu.edu.cn (T.W.); mywang@bjmu.edu.cn (M.-Y.W.); wangxw@bjmu.edu.cn (X.-W.W.); siyue.wang@pku.edu.cn (S.-Y.W.); Jiating@pku.edu.cn (J.-T.W.); yuhh@pku.edu.cn (H.Y.); 2 Medical Informatics Center, Peking University, Beijing 100191, China

**Keywords:** healthcare utilization, cost analysis, diabetes, complication

## Abstract

We aimed to provide reliable regression estimates of expenditures associated with various complications in type 2 diabetics in China. In total, 1,859,039 type 2 diabetes patients with complications were obtained from the Beijing Medical Claim Data for Employees database from 2008 to 2016. We estimated costs for complications using a generalized estimating equation model adjusted for age, sex, and the incidence of various complications. The average total cost for diabetic patients with complications was 17.12 thousand RMB. Prescribed drugs accounted for 63.4% of costs. We observed a significant increase in costs in the first year after the onset of complications. Compared with costs before the incidence of complications, the additional costs per person in the first year and >1 year after the event would be 10,631.16 RMB and 1150.71 RMB for cardiovascular disease, 1017.62 RMB and 653.82 RMB for cerebrovascular disease, and 301.14 RMB and 624.00 RMB for kidney disease, respectively. The estimated coefficients for outpatient visits were relatively lower than those of inpatient visits. Complications in diabetics exert a significant impact on total healthcare costs in the first year of their onset and in subsequent years. Our estimates may assist policymakers in quantifying the economic burden of diabetes complications.

## 1. Introduction

Diabetes is a common challenge faced by both developed and developing countries and imposes a heavy burden on national economies and medical systems. According to the International Diabetes Federation, the disease affected 451 million adults worldwide in 2017, and global healthcare costs for diabetics were estimated to be USD 850 billion [1]. Moreover, the economic costs of diabetes are increasing rapidly in developing countries, particularly in Asia [2]. In many Asian countries, people tend to develop diabetes at younger ages and suffer longer with complications of diabetes than people in other regions, which may cause a heavier disease burden [3,4,5]. As the largest developing country, China has a growing burden of diabetes [6]. Studies have shown that the incidence of various complications may further increase the socio-economic burden of diabetes, focusing attention on the related costs of diabetes complications [7,8,9].

As more diabetes treatment options emerge, economic evaluations of the related costs become important. Previous systematic disease models such as those of the Centers for Disease Control and Prevention/RTI International and the UK Prospective Diabetes Study are available to assess the cost-effectiveness of disease intervention programs [10,11]. These models can track patients over a long period and predict the development of complications. However, because of the differing cost characteristics between countries, these models may not be suitable for China [12]. Thus, a more reliable means of estimating costs is required.

To date, only a few studies have investigated the medical costs of diabetes complications in China, and these studies only analyzed the average cost of a specific complication or a total complication based on a single or several hospitals [8,9,13]. In the present study, we offer a fuller and more representative picture of the association between healthcare costs and complications in patients with type 2 diabetes in Beijing, China. Moreover, it is the first study in China to use regression methods to estimate the short- and medium-term costs of type 2 diabetes-related complications. These estimates can assist clinicians and policymakers in quantifying the economic burden of diabetes complications.

## 2. Materials and Methods

### 2.1. Data Source

The health data used in this study were extracted from the Beijing Medical Claim Data for Employees (BMCDE) database, which was described previously [14]. All employers in the urban areas of Beijing, including government agencies and institutions, state-owned enterprises, private businesses, social organizations, and other private units and their employees and retirees are obligated to enroll in this database. By the end of 2017, more than 20 million individuals were listed in the database, covering nearly 90% of Beijing’s permanent residents. Medical information recorded in the BMCDE database includes patient characteristics (age and sex), treatments, clinical diagnoses coded using the International Classification of Diseases, 10th Revision (ICD-10), and costs. Each person is assigned a unique, pseudonymous identification number. Our data were collected for an administrative purpose without any personal identifiers; therefore our study was exempted from ethics committee review.

### 2.2. Study Sample

Patients with type 2 diabetes were identified using the E11 code in ICD-10 and/or prescriptions for hypoglycemic drugs or insulin. Data from 1 January 2008 to 31 December 2016 were included. Diabetic complications consisted primarily of acute complications such as diabetic ketoacidosis and diabetic coma and chronic complications (peripheral vascular disease, kidney disease, ocular disorders, cerebrovascular disease, cardiovascular disease, and neuropathy). Table S1 in Supplementary Material 1 lists the ICD-10 codes for these diseases. Onset of diabetic complications was determined by the date the individual was first diagnosed. We took age at diagnosis as onset age. Individuals with diabetes who were aged ≥18 years and had at least one hospital visit were included in this study. We excluded type 2 diabetic patients with pre-existing complications by a washout period of 2 years (2008–2009). In addition, patients who had died during the follow-up period were excluded from this study. Patient records were included in the present study if information was available in the database regarding their age, sex, and total medical costs (including costs for the hospital bed, prescribed drugs, laboratory tests, medical examinations, surgery, and radiology).

### 2.3. Statistical Analysis

Chinese currency experienced sustained inflation between 2008 and 2016. To better reflect the real monetary value, all costs were expressed in 2008 China Yuan (RMB) by dividing a monetary time series of the consumer price index in Beijing (Table S2 in Supplementary Material 1). Annual consumer price index data in Beijing were obtained from the Beijing Municipal Bureau of Statistics (http://tjj.beijing.gov.cn/, last retrieved on 27 February 2021).

To ensure the reliability of the statistical analysis, each step was double-analyzed. We also checked the quality and plausibility of the data, removing outliers such as negative medical costs [15]. We used the calendar year as the observation period, from 2 years before the onset of complications to 6 years after their onset. In accordance with previous studies, we estimated the impact of complications in two time periods using the requirements of the implementation of costs in diabetes models: <1 year after the incidence of complications and >1 year after the incidence of complications [12,16]. We hypothesized that later stages might reflect the continuing impact of complications, so we included patients who had experienced such complications in separate dummy variables, kept them in the same group, and followed up until death or the end of 2016.

We used descriptive analyses for demographic and complication characteristics of type 2 diabetic patients. The consumer price index-adjusted average total medical costs were calculated by year and type of complication. Categorical variables were expressed as percentages. We used a generalized estimating equations (GEE) model with a Gaussian distribution to explain the nonindependence of observations within each subject during the study period (for details, see Supplementary material 2). The GEE method is commonly used in analyzing longitudinal data when the population-averaged effect is of interest. Our study was based on the city-wide, large-scale claim data of diabetic patients. As reported previously, if the sample size was sufficiently large and the proportion of cases with zero costs was relatively small, we could assume that the sample mean was near normality [17,18]. Furthermore, the GEE model with a normal distribution showed better model fit based on the mean square error and residual plot compared with a gam-ma-based GEE model when ¥1 was assigned for patients with zero costs. In addition, the normal distributions displayed good properties for quantification of probabilistic uncertain-ties and interpretability of results. Thus, we used the GEE model based on the normal distribution in this study. The GEE model controlled for sex and age. We also compared outpatient and inpatient costs. *p* < 0.05 was considered statistically significant. Statistical analyses were performed using SAS version 9.4 (SAS Institute Inc., Cary, NC, USA).

## 3. Results

### 3.1. Demographics

Table 1 summarizes the demographic characteristics of diabetic patients included in the study. A total of 1,859,039 patients aged over 18 years were included. Of these, 53.03% were female. The average age was 65.01 years. The majority of patients were aged 55–64 years (30.58%), followed by patients aged 65–74 years (27.46%). Approximately 41.00% had only one complication, and this cohort had a higher proportion of males (43.16%). Approximately 30.20%, 17.59%, and 11.21% of patients had two, three, and four or more complications, respectively.

### 3.2. Descriptive Analysis

Table 2 lists the types of complications observed in the study cohort. Approximately 40% (738,589) of patients had cardiovascular disease, which was the most common complication. In descending order of prevalence, the other complications were cerebrovascular disease, kidney disease, ocular disorders, neuropathy, peripheral vascular disease, and diabetic coma.

**Figure 1:**
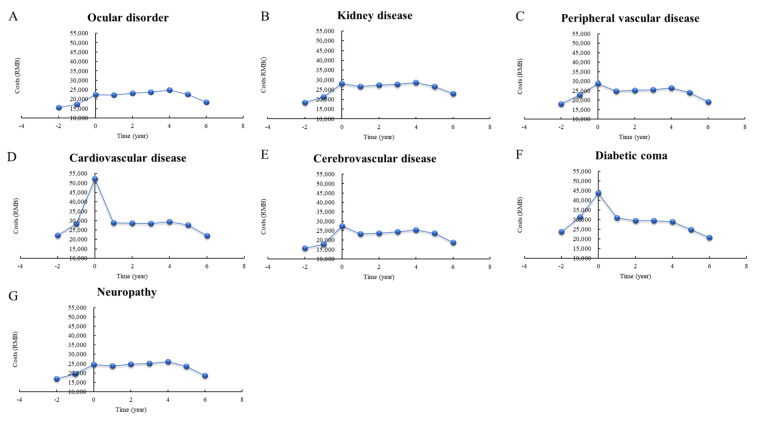
Distribution of direct total medical costs per person before and after the incidence of complications, stratified by year. The x-axis shows the time period for the complication. “−2” means >2 years prior to the event; “−1” means <1 year before the event; “0” means <1 year after the event; “1” means 1−2 years after the event.

Figure 1 shows the progress of direct total medical costs per person before, during, and after the incidence of complications. The distribution of medical costs per person in the first year after the event indicates a significant peak for complications such as peripheral vascular disease (28.76 thousand RMB), cardiovascular disease (52.23 thousand RMB), cerebrovascular disease (27.45 thousand RMB), and diabetic coma (43.73 thousand RMB) followed by marked decreases in the following years. For other events, the costs increased after the event, but increased slowly or remained stagnant in the following 4 years; these included ocular disorders, kidney disease, and neuropathy.

**Figure 2:**
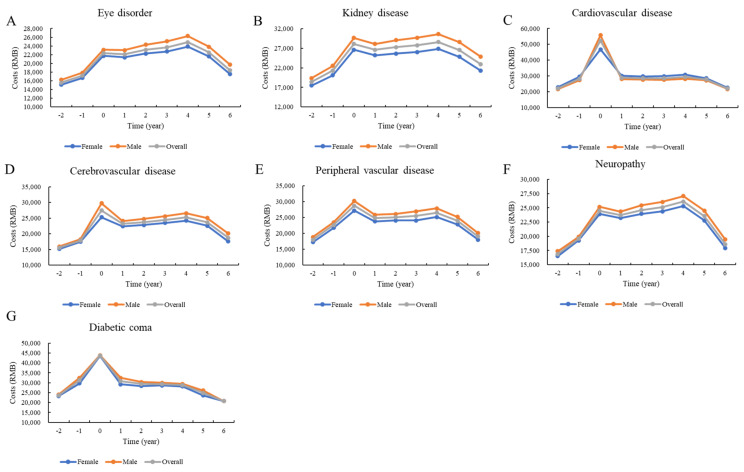
Distribution of direct total costs before and after the incidence of complications, stratified by year and sex.

**Figure 3:**
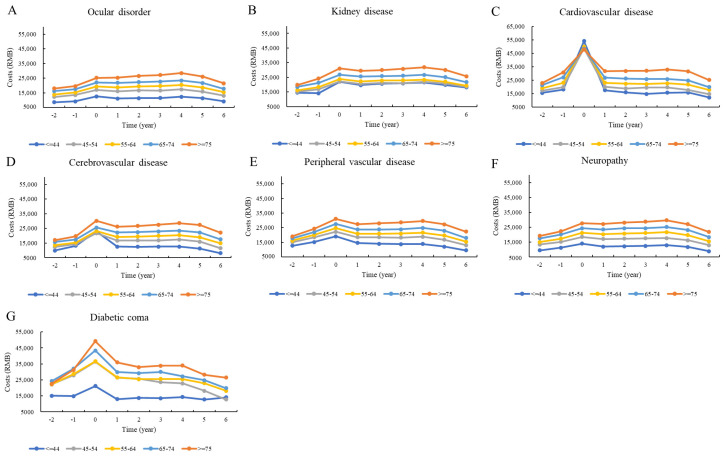
Distribution of direct total costs before and after the incidence of complications, stratified by year and age.

**Figure 4:**
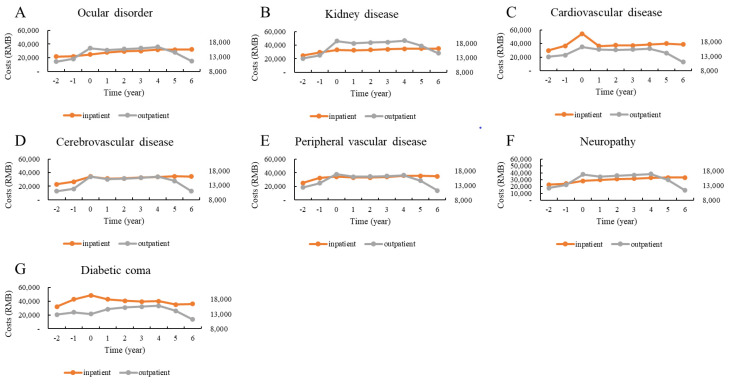
Distribution of direct total costs before and after the incidence of complications, stratified by year and type of hospital visit.

Figure 2, Figure 3 and Figure 4 show the distributions of direct total medical costs per person adjusted by sex, age, and type of hospital visit. Generally, the sex-specific and age-specific distributions of medical costs of patients were similar to those of the overall distribution. The direct total medical costs were much higher in male patients than in female patients (Figure 2). Older patients had higher medical expenses. However, patients under 45 years paid the most money to hospitals in the first year after the incidence of cardiovascular disease (Figure 3). There were significant differences in costs between inpatient and outpatient departments. For patients with cardiovascular and cerebrovascular diseases, the direct total medical costs for both inpatient and outpatient visits were highest in the first year after the incidence of the complication. While the costs for patients with ocular disorders and neuropathy were higher for outpatient visits, the costs for patients with diabetic coma were highest for inpatient visits for the first year after the incidence of the coma (Figure 4).

### 3.3. Regression Analysis

Table 3 shows the estimated coefficients obtained from the GEE model for the costs for inpatient visits. After adjusting for other risk factors, older patients would have additional costs of from 2173.74 RMB (patients aged 45–54 years) to 6333.84 RMB (patients aged over 74 years) compared with patients aged under 45. Men’s costs would be 2236.33 RMB higher than women’s costs. After adjusting for other complications, compared with costs before the incidence of complications, the additional costs per person in the first year and >1 year after the event would be 10,631.16 RMB and 1150.71 RMB for cardiovascular disease, 1017.62 RMB and 653.82 RMB for cerebrovascular disease, and 301.14 RMB and 624.00 RMB for kidney disease, respectively. While patients with diabetic coma would have significantly higher medical costs in the year of the event, patients with ocular disorders and neuropathy would have additional costs of 1423.16 RMB and 883.90 RMB, respectively, in the following years. The estimated coefficients for outpatient visits were relatively lower than those of inpatients visits. For most complications, patients would have additional costs in the following years. Sex and age differences in costs for outpatient visits followed the same patterns as costs for inpatient visits (Table 4).

## 4. Discussion

To the best of our knowledge, this is the first city-wide study drawing a comprehensive picture of the association between diabetes complications and healthcare costs in China. This study provided adequately specified information on the costs of type 2 diabetes in patients with acute or chronic microvascular and macrovascular complications. We found that diabetic patients had an increase in total medical costs during the first year of the incidence of the complication, and the expenses associated with some complications continued to rise in subsequent years. In addition, we found differences in the cost of complications between the sexes and among age groups.

Our findings of rising costs after the onset of complications were consistent with those of previous studies. A cross-sectional study in rural southeast China reported that the mean total cost for patients with one complication was USD 1399, and the total hospitalization and outpatient costs of patients with complications were 83.55% and 38.46% higher, respectively, than those of patients without complications [19]. A study that collected data from 38 tertiary hospitals in Beijing between 2006 and 2010 showed that diabetic complications increased hospitalization costs and length of stay considerably in patients with type 2 diabetes; the median total hospitalization cost for patients without complications was 6349.72 RMB, while the median total hospitalization cost for patients with multiple complications was 12,177.62 RMB (*p* < 0.001) [7]. Similarly, a cross-sectional survey of 15 hospitals in urban China also reported that complications significantly increased outpatient visits of type 2 diabetics (odds ratio: 1.064, *p* < 0.001) [13]. The gap in the specific costs between our estimates and those of previous studies may be attributable to the more severe complications included in our study. Moreover, differences in region, hospital levels, and sample size may have also been involved. Non-Chinese studies have reported similar findings. For example, a study based on German national claim data and a multi-country comparative analysis both reported that diabetic complications increase hospital use and costs significantly and can exert an economic burden on the healthcare system [10,12]. Furthermore, our follow-up observations are consistent with those of studies that concluded that the costs of eye diseases and kidney disease will continue to rise over the next few years [20,21,22].

Our subgroup analyses indicated that the cost of complications was higher in men, but perhaps this difference may have been related to age distribution, as the men in our study were, on average, 2.8 years older than the women. Moreover, some diseases displayed sex-based differences in severity and subtype distribution [23,24,25]. This finding is consistent with those of most previous studies, but we also found contrasting evidence [12,26]. Despite differences in costs and healthcare systems between countries, we tended to be cautious about explaining sex differences. In addition, we also found that the elderly (>75 years) had the highest medical costs for diabetic complications, a finding consistent with those of previous studies [27,28,29].

We also compared outpatient and inpatient costs using the GEE model. The cost of most complications increased significantly after the initial diagnosis of the disease. It is important to note that a significant increase in outpatient costs was only observed one year after the initial diagnosis of the complication; perhaps the symptoms are more severe in the first year of diagnosis, leading to more hospitalization [30,31]. After one year, because the number of hospital beds is limited, some patients who successfully controlled their disease were followed-up in the outpatient department, leading to an increase in outpatient services [30,31,32,33]. In addition, we found that the cost of peripheral vascular disease did not increase significantly in the model, perhaps because it is frequently accompanied by cardiovascular diseases [34,35]. Adjusting for cardiovascular diseases affected the estimate for peripheral vascular disease.

Our study has several strengths. First, we used regression analysis to reliably estimate the costs associated with various complications in type 2 diabetics, adjusting for sex, age, and other complications. Second, this study is based on medical insurance data from Beijing, providing a large and representative sample of the costs of diabetes and ensuring the stability of the results. The large sample also enabled us to assess some less-prevalent complications such as diabetic ketoacidosis and diabetic coma. Third, we included patients who had been insured throughout the study period and had annual medical records, maintaining the integrity of cost-tracking. However, some potential limitations should be noted. First, the data in this study are from the BMCDE database, which does not include individuals under 18 years or temporary or seasonal residents. Thus, the study did not cover all of Beijing’s population, and we were unable to analyze the related costs for children. Second, as in previous studies using the large administrative health database, we could not fully explore other related covariates like BMI, glucose level, smoking, and functional status, which are considered to be associated with healthcare costs. Future studies are needed to investigate the effect of these covariates on costs of complications. Third, we only know the initial diagnosis time of the complications rather than the true onset time, which should be evaluated by prospective clinical studies.

## 5. Conclusions

In conclusion, complications in type 2 diabetics had a significant impact on total medical costs, especially in the first year of the events. Male patients, the elderly (>75 years old), and patients who required hospitalization were the groups with the highest medical costs. Our study provides new evidence for investigating diabetes costs and may promote future research in the field of diabetes modeling in China.

## Figures and Tables

**Table 1:** Characteristics of diabetic patients.

Variable	Overall	Female	Male
	(*n* = 1,859,039)	(*n* = 985,797)	(*n* = 873,242)
Sex (%)		53.03	46.97
Age, years, mean	65.01	64.40	65.70
Age group, number (%)			
≤44	79,407 (4.27)	36,606 (3.71)	42,801 (4.90)
45–54	335,158 (18.03)	197,010 (19.98)	138,148 (15.82)
55–64	568,559 (30.58)	313,664 (31.82)	254,895 (29.19)
65–74	510,500 (27.46)	258,333 (26.21)	252,167 (28.88)
≥75	365,415 (19.66)	180,184 (18.28)	185,231 (21.21)
Number of complications			
1	762,130 (41.00)	385,273 (39.08)	376,857 (43.16)
2	561,424 (30.20)	305,143 (30.95)	256,281 (29.35)
3	327,087 (17.59)	180,665 (18.33)	146,422 (16.77)
≥4	208,398 (11.21)	114,716 (11.64)	93,682 (10.73)

**Table 2:** Proportions of diabetic patients by type of complications.

Variables	No. (%)
Type of complications	
Cardiovascular disease	738,589 (39.73)
Cerebrovascular disease	571,915 (30.76)
Kidney disease	345,728 (18.60)
Eye disorder	230,866 (12.42)
Neuropathy	191,710 (10.31)
Peripheral vascular disease	186,780 (10.05)
Diabetic coma	9221 (0.50)

**Table 3:** Effects of complications on direct total medical costs per person for inpatient visits in generalized estimating equation normal regression.

**Variable**	**Coefficient Estimate** (**Standard Error**)	
Sex: female (Ref: male)	−2236.33 *** (28.66)	
Age group (Ref: ≤44 years), years		
45–54	2173.74 *** (56.14)	
55–64	3715.05 *** (54.52)	
65–74	5582.47 *** (58.31)	
≥75	6333.84 *** (59.20)	
	**Coefficient Estimate** (**Standard Error**)	
Event/condition (Ref: *n*)	<1 year after the event	>1 year after the event
Neuropathy	−16.14 (115.09)	883.90 *** (91.01)
Ocular disorders	−503.66 *** (106.65)	1423.16 *** (93.39)
Kidney disease	304.14 ** (108.43)	624.00 *** (83.08)
Cardiovascular disease	10,631.16 *** (125.74)	1150.71 *** (86.10)
Peripheral vascular disease	−748.48 *** (129.33)	−1033.74 *** (114.89)
Cerebrovascular disease	1017.62 ** (74.56)	653.82 *** (70.28)
Diabetic coma	3135.64 *** (492.21)	−1031.60 ** (436.63)

Ref, reference. ** *p* < 0.01; *** *p* < 0.001.

**Table 4:** Effects of complications on direct total medical costs per person for outpatient visits in generalized estimating equation normal regression.

**Variable**	**Coefficient Estimate** (**Standard Error**)	
Sex: female (Ref: male)	−29.78 *** (0.39)	
Age group (Ref: ≤44 years), years		
45–54	46.02 *** (0.80)	
55–64	57.73 *** (0.80)	
65–74	75.86 *** (0.85)	
≥75	73.24 *** (0.88)	
	**Coefficient Estimate** (**Standard Error**)	
Event/condition (Ref: *n*)	<1 year after the event	>1 year after the event
Neuropathy	−6.28 *** (0.64)	51.89 *** (0.76)
Ocular disorders	−11.70 *** (2.30)	89.07 *** (3.31)
Kidney disease	−23.53 *** (0.76)	52.18 *** (0.97)
Cardiovascular disease	−3.34 ** (1.20)	29.79 *** (1.51)
Peripheral vascular disease	−14.07 *** (0.77)	27.02 *** (0.90)
Cerebrovascular disease	−11.28 ** (0.40)	31.84 *** (0.48)
Diabetic coma	−30.39 *** (4.36)	25.54 *** (5.59)

Ref, reference. ** *p* < 0.01; *** *p* < 0.001.

## Data Availability

Restrictions apply to the availability of these data. Data was obtained from the administrative department of China’s health and medical system and are available with the permission of the administrative department.

## Supplementary Materials

The following are available online at https://www.mdpi.com/article/10.3390/ijerph18073693/s1, Supplementary file 1: The ICD-10 used to diagnose the complications and the consumer price index-adjusted costs and Supplementary file 2: The parameters of the generalized estimating equations (GEE) model.
