# Supplementary material for: Healthcare Costs Associated with Complications in Patients with Type 2 Diabetes among 1.85 Million Adults in Beijing, China
Source: Int J Environ Res Public Health. 2021 Apr 1;18(7):3693. doi: 10.3390/ijerph18073693 (PMC8036594; doi:10.3390/ijerph18073693)
Supplement: Supplementary file 1 [file ijerph-18-03693-s001.zip › Supplementary material 1.docx]

Supplementary material 1

Table 1 The International Classification of Disease Tenth Revision (ICD-10) used to diagnose the complications

| Complication | ICD-10 |
| --- | --- |
| Acute complications  (diabetic ketoacidosis and diabetic coma) | E15  E11.001  E11.101  E11.102  E14.102  E14.103  E14.001  E14.002  E14.003 |
| Peripheral neuropathy | E14.401+  E14.402+  E14.403+  E14.404+  E14.405+  E14.406+  E14.407+  E14.408+  E14.608+  E14.609+ |
| Diabetic kidney disease | E14.201+  E14.202+  E14.203+  N18.902  N18.903  N18.905  N17.901  N17.902  N17.903  N17.904  N99.001  N19 02  N19 03  N18.807 |
| Ocular disorders | E14.301  E14.302+  E14.303+  E14.304+  E14.305+ |
| Cerebrovascular disease | I67.805  I69.803  I60.802  I61.001  I61.003  I61.301  I61.303  I61.401  I62.001  I61.002  I61.101  I61.302  I61.304  I61.501  I61.902  I62.102  I69.101  I63.801  I63.902  I63.904  I69.301  I63.901  I63.903  I64 02  I64 04 |
| Cardiovascular disease | I48 02  I48 04  I48 06  I21.001  I21.002  I21.003  I21.101  I21.102  I21.103  I21.202 - I21.212  I21.301 - I21.304  I21.401  I21.402  I21.902  I22.913  I21.002  I21.004  I21.901  I21.907  I21.912  I49.002  I50.001  I50.003  I50.101  I50.105  I50.107  I50.904  I50.912  I97.101  I50.004  I50.102  I50.106  I50.903  I50.905  I50.911  I97.104  I97.106  I25.201  I25.203  I25.205  I25.207  I25.209  I25.211  I25.202  I25.204  I25.206  I25.208  I25.210  I25.212  I71.002  I71.001  I71.201  I71.203  I71.205  I71.302  I71.402  I71.901  I71.002  I71.101  I71.204  I71.301  I71.902 |
| Peripheral vascular disease | E14.503  L97 01  L88 01  I21.906  I26.901  I51.302  I63.301  I65.002  I65.102  I66.903  I73.101  I74.002  I74.101  I74.301  I74.305  I74.307  I74.402  I74.504  I74.802  I74.804  I74.808  I74.902  I80.102  I80.204  I80.206  I80.801  I80.803  I80.805  I80.902  I82.201  I82.203  I82.302  I82.802  I82.804  I82.806  I82.808  I82.901  I84.001  I84.301  I21.905  I26.902  I51.303  I63.401  I65.101  I65.204  I65.206  I66.301  I66.801  I66.902  I66.904  I74.003  I74.102  I74.202  I74.302  I74.306  I74.401  I74.501  I74.503  I74.803  I74.805  I74.807  I74.901  I80.101  I80.201  I80.205  I80.301  I80.303  I80.802  I82.101  I82.202  I82.301  I82.801  I82.803  I82.805  I82.807  I82.809  I82.902  E11.501  E14.501  A48.001 |

Table 2 Consumer price index-adjusted costs

| Year | Consumer price index (%) | Unadjusted costs | Adjusted costs |
| --- | --- | --- | --- |
| 2008 (base year) | 105.1 | Y1 | Y1′=Y1 |
| 2009 | 98.5 | Y2 | Y2′=Y2/98.5% |
| 2010 | 102.4 | Y3 | Y3′=Y3/102.4%/98.5% |
| 2011 | 105.6 | Y4 | Y4′=Y4/105.5%/102.4%/98.5% |
| 2012 | 103.3 | Y5 | Y5′=Y5/105.5%/102.4%/98.5%/103.3% |
| 2013 | 103.3 | Y6 | Y6′=Y6/105.5%/102.4%/98.5%/103.3%/101.3% |
| 2014 | 101.6 | Y7 | Y7′=Y7/105.5%/102.4%/98.5%/103.3%/101.3%/101.6% |
| 2015 | 101.8 | Y8 | Y8′=Y8/105.5%/102.4%/98.5%/103.3%/101.3%/101.6%/101.8% |
| 2016 | 101.4 | Y9 | Y9′=Y9/105.5%/102.4%/98.5%/103.3%/101.3%/101.6%/101.8%/101.4% |

Table 3 The proportion of medical expenses of diabetes patients with different complications in Beijing, China (%)

| Type of complication | Type of patient | Cost of prescribed drugs | Examination and treatment costs | Cost of medical materials | Costs for the hospital bed | Other |
| --- | --- | --- | --- | --- | --- | --- |
| Eye disorder | inpatient | 35.92 | 32.23 | 26.31 | 2.37 | 3.17 |
|  | outpatient | 79.34 | 16.00 | 2.58 | 0.00 | 2.08 |
| Kidney disease | inpatient | 37.56 | 32.62 | 24.37 | 2.41 | 3.03 |
|  | outpatient | 77.19 | 17.70 | 3.01 | 0.00 | 2.10 |
| Cardiovascular disease | inpatient | 35.28 | 30.72 | 28.62 | 2.27 | 3.11 |
|  | outpatient | 79.84 | 15.57 | 2.29 | 0.00 | 2.29 |
| Cerebrovascular disease | inpatient | 37.79 | 32.00 | 24.62 | 2.44 | 3.15 |
|  | outpatient | 81.25 | 14.34 | 2.17 | 0.00 | 2.24 |
| Peripheral vascular disease | inpatient | 37.68 | 31.08 | 25.75 | 2.37 | 3.13 |
|  | outpatient | 78.83 | 16.29 | 2.71 | 0.00 | 2.17 |
| Neuropathy | inpatient | 36.51 | 32.09 | 25.82 | 2.42 | 3.16 |
|  | outpatient | 81.74 | 14.02 | 2.16 | 0.00 | 2.07 |
| Diabetic coma | inpatient | 44.37 | 34.23 | 16.42 | 2.53 | 2.45 |
|  | outpatient | 77.89 | 17.18 | 2.79 | 0.00 | 2.14 |
