# Supplementary material for: Healthcare Costs Associated with Complications in Patients with Type 2 Diabetes among 1.85 Million Adults in Beijing, China
Source: Int J Environ Res Public Health. 2021 Apr 1;18(7):3693. doi: 10.3390/ijerph18073693 (PMC8036594; doi:10.3390/ijerph18073693)
Supplement: Supplementary file 1 [file ijerph-18-03693-s001.zip › Supplementary material 2.docx]

Supplementary material 2

Suppose $y_{\mathrm{ij}}$, j = 1,…,n, i = 1,…,K, K represent the jth response of the ith subject, which has a vector of covariates $X_{\mathrm{ij}}$ . There are $n_{i}$measurements on subject i, and the maximum number of measurements per subject is T.

Suppose the responses of the ith subject be $Y_{i}$ = [$y_{i1},\ldots, y_{in_{i}}$]’ with corresponding means $\mu_{i}$ = [$\mu_{i1},\ldots, \mu_{in_{i}}$]’. For generalized linear models, the marginal mean $\mu_{\mathrm{ij}}$ of the response $y_{\mathrm{ij}}$ is related to a linear predictor through a link function $g\left( \mu_{\mathrm{ij}} \right) = {X'}_{\mathrm{ij}}\beta$, and the variance of $y_{\mathrm{ij}}$ depends on the mean through a variance function $\nu\left( \mu_{\mathrm{ij}} \right)$.

The model is therefore noted as follows:

$E\left( y_{\mathrm{ij}} \right)=\mu_{\mathrm{ij}}$,

$$g\left( \mu_{\mathrm{ij}} \right)=\beta_{0}+\beta_{1}X_{i1}+\beta_{k2}X_{ki2}+\beta_{mn3}X_{mnij3}+e_{\mathrm{ij}}$$

where:

i = patient i

k = age group k

m = comorbidity m

j = observation j

n = time period n for a comorbidity

$\mu_{\mathrm{ij}}$ = CPI-adjusted total medical costs for patient i and observation j

$\beta_{0}$ = coefficient for the intercept

$\beta_{1}$ = coefficient for sex

$X_{i1}$ = dummy variable for sex

$\beta_{k2}$ = coefficient for age group k

$X_{ki2}$ = dummy variables for the age group

$\beta_{m3}$ = coefficient for time period n for comorbidity m

$X_{mnij3}$ = dummy variables for time period n for comorbidity m

$e_{\mathrm{ij}}$ = error term for patient i, observation j
